# Regulation of T Cell Differentiation and Function by EZH2

**DOI:** 10.3389/fimmu.2016.00172

**Published:** 2016-05-03

**Authors:** Theodoros Karantanos, Anthos Christofides, Kankana Bardhan, Lequn Li, Vassiliki A. Boussiotis

**Affiliations:** ^1^Division of Hematology-Oncology, Beth Israel Deaconess Medical Center, Harvard Medical School, Boston, MA, USA; ^2^Department of Medicine, Beth Israel Deaconess Medical Center, Harvard Medical School, Boston, MA, USA; ^3^General Internal Medicine Section, Boston Medical Center, Boston University School of Medicine, Boston, MA, USA; ^4^Beth Israel Deaconess Cancer Center, Harvard Medical School, Boston, MA, USA

**Keywords:** T cells, T cell differentiation, T cell activation, tumor immunity, EZH2

## Abstract

The enhancer of zeste homolog 2 (EZH2), one of the polycomb-group proteins, is the catalytic subunit of Polycomb-repressive complex 2 (PRC2) and induces the trimethylation of the histone H3 lysine 27 (H3K27me3) promoting epigenetic gene silencing. EZH2 contains a SET domain promoting the methyltransferase activity, while the three other protein components of PRC2, namely EED, SUZ12, and RpAp46/48, induce compaction of the chromatin permitting EZH2 enzymatic activity. Numerous studies highlight the role of this evolutionary conserved protein as a master regulator of differentiation in humans involved in the repression of the homeotic gene and the inactivation of X-chromosome. Through its effects in the epigenetic regulation of critical genes, EZH2 has been strongly linked to cell cycle progression, stem cell pluripotency, and cancer biology, being currently at the cutting edge of research. Most recently, EZH2 has been associated with hematopoietic stem cell proliferation and differentiation, thymopoiesis and lymphopoiesis. Several studies have evaluated the role of EZH2 in the regulation of T cell differentiation and plasticity as well as its implications in the development of autoimmune diseases and graft-versus-host disease (GVHD). The aim of this review is to summarize the current knowledge regarding the role of EZH2 in the regulation of the differentiation and function of T cells focusing on possible applications in various immune-mediated conditions, including autoimmune disorders and GVHD.

## Introduction

The enhancer of zeste homolog 2 (EZH2), one of the polycomb-group (PcG) proteins, is the catalytic subunit of Polycomb-repressive complex 2 (PRC2) and induces the trimethylation of the histone H3 lysine 27 (H3K27me3) promoting epigenetic gene silencing (1, 2). EZH2 contains a SET domain promoting the methyltransferase activity, while the three other protein components of PRC2, namely EED, SUZ12, and RpAp46/48, induce compaction of the chromatin permitting the enzymatic activity of EZH2 (3). Numerous studies highlight the role of this evolutionary conserved protein as a master regulator of differentiation in humans involved in the repression of the homeotic (Hox) gene and the inactivation of X-chromosome (3–6).

Through its implications in the epigenetic regulation of critical genes, EZH2 has been strongly linked to cell cycle progression (7), stem cell pluripotency (8, 9), and cancer biology being currently at the cutting edge of research. At the molecular level, maintaining proper levels of EZH2 is critical for the normal function of cells, while its aberrant expression has been implicated in the induction of cell proliferation and oncogenesis (10, 11). Interestingly, EZH2 is aberrantly overexpressed in a variety of neoplasms compared to normal tissues (11, 12) and is currently the subject of intense research for the better understanding of cancer biology. Thus, EZH2 is currently evaluated as a new biomarker and a novel target for cancer therapy.

Most recently, EZH2 has been associated with hematopoietic stem cell proliferation and differentiation (13, 14), thymopoiesis (14), and lymphopoiesis (15). Numerous studies have evaluated the role of EZH2 in the regulation of T cell differentiation and plasticity as well as its implications in the development of autoimmune diseases and graft-versus-host disease (GVHD). Given the currently evolving development of EZH2 inhibitors for human malignancies, the introduction of these inhibitors as modulators of immune-mediated diseases and activation of the immune system in patients with cancers is a promising opportunity.

## EZH2 and Regulation of T Cell Differentiation

### T Cell Lineage Commitment

The exposure of naïve T helper (Th) cells to an antigen in the context of distinct extracellular microenvironment queues leads to differentiation into the effector lineages namely Th1, Th2, and Th17 expressing distinct cytokines genes (16, 17). It is known that Th1 lineage produces IFN-γ and is implicated in anti-microbial and anti-viral immunity, while Th2 cells produce IL-4 and are involved in immunity against parasites as well as in the pathogenesis of allergic reactions through the activation of B cells and the subsequent production of IgE antibodies inducing the activity of mast cells (18). Th17 cells produce mainly IL-17 and IL-22 and are critical for the protection against certain extracellular pathogens, such as *Candida albicans* and *Staphylococcus aureus*, but are also involved in the pathophysiology of autoimmune conditions, such as inflammatory bowel disease (19, 20). The differentiation of naïve Th cells is promoted by a variety of cytokines produced in the setting of localized or systemic inflammation and is associated with a pattern of expression of lineage-specific transcription factors. Particularly, IL-12 promotes the polarization toward Th1 lineage associated with the induction of the T-bet transcriptional factor, while IL-4 potentiates the differentiation toward Th2 lineage that is connected with induction of the GATA-3 transcription factor (21). For the differentiation of Th17 cells, TGF-beta, IL-6, and activation of STAT3 and RORγ transcription factors have indispensable roles (22).

The expression of the transcription factor forkhead box P3 (FOXP3) defines a subset of CD4^+^ cells with inhibitory functions called T regulatory cells (Tregs). Natural Tregs differentiate in the thymus maintaining and expanding the FOXP3 expression with the involvement of IFN-γ, TGF-beta, and IL-2, while naïve FOXP3^−^ cells can differentiate directly in the periphery to FOXP3^+^ *de novo* induced Tregs through the exposure to antigens with the involvement mainly of TGF-beta. Natural Tregs expressing T-bet transcription factor are known to suppress the function and expansion of Th1 cells, while Treg expressing IRF4 and Stat3 are critical for the inhibition of Th2 and Th17 cells, respectively (23). These conclusions highlight the importance of Tregs development and maintenance, which is controlled by numerous factors, including cytokines, transcription factors, and epigenetic regulators.

### EZH2 and T Cell Differentiation

Recent studies highlight the role of EZH2 in the differentiation and plasticity of T cells and introduce a new critical player with possible therapeutic implications. Data presented in an early study by Raaphorst et al. supported that the expression of the PcG proteins BMI-1 and EZH2 in mature peripheral T cells is mutually exclusive and associated with their proliferation status (24). Of note, particular patterns of EZH2 and BMI-1 expression characterize different stages of T cells differentiation in the thymus, suggesting a critical regulatory role of PcG proteins in lymphopoiesis through the stabilization of gene expression patterns that will lead to a lineage choice (24).

**Figure 1:**
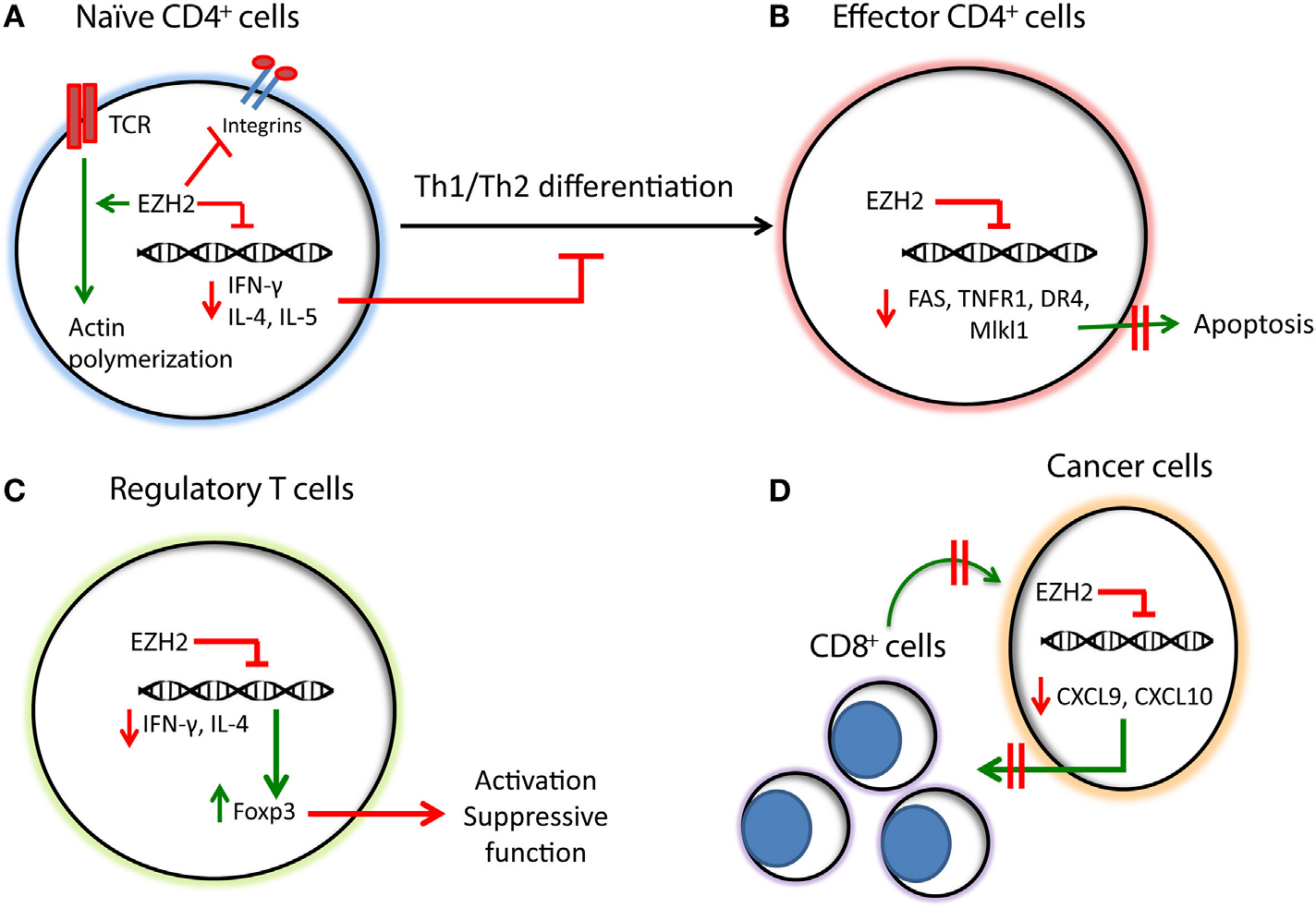
**Summary of the functions of EZH2 in T cells, regulatory T cells, and cancer-mediated immune regulation**. **(A)** EZH2 downregulates the expression of Th1/Th2 cytokines, such as IFN-γ and IL-4/IL-5, respectively, inhibiting the Th1/Th2 differentiation of naïve CD4^+^ T cells. EZH2 inhibits integrin-dependent migration of T cells while promoting TCR-dependent actin polymerization. **(B)** In effector CD4^+^ T cells EZH2 inhibits the expression of apoptotic molecules, such as FAS, TNFR1, DR4, and Mlkl1, promoting their survival and sustaining the immune responses. **(C)** EZH2 downregulates the expression of IFN-γ and IL-4, while promoting the expression of Foxp3 and the activation of T regulatory cells. **(D)** In cancer cells, EZH2 inhibits the expression of Th1 chemokines, such as CXCL9 and CXCL10 thereby compromising the trafficking of CD8^+^ T cells, which mediate cytotoxic effects on cancer cells and promote anti-tumor function.

Subsequent studies using EZH2-deficient T cells determined that EZH2 has a key role in the differentiation and plasticity of Th1 and Th2 T cells and a mandatory role on the survival of T effector cells. Specifically, Zhang et al. evaluated the role of EZH2 expression in the differentiation of naïve CD4^+^ T cells and the survival of effector cells, using a model of transgenic EZH2^fl/fl^CD4-Cre mice for T cell-specific EZH2 deletion (25). These studies revealed that the proportion of effector Th0, Th1, Th2, and Th17 but not iTreg cells in the EZH2^fl/fl^CD4-Cre mice were decreased by day 6 after birth (25). Interestingly, the study showed that EZH2 deletion was associated with upregulation of IFN-γ and IL-10 under Th0 conditions, enhanced induction of IFN-γ under Th1 conditions and increased expression of IL-10 under Th2 conditions (25). Similarly, Yang et al. showed that EZH2-deficient CD4^+^ T cells produce higher amounts of IFN-γ, IL-13, and IL-17 in Th1, Th2, and Th17 conditions, respectively, compared to control T cells, suggesting that EZH2 suppresses the expression of lineage signature cytokines (26). Together these studies indicate that EZH2 has an inhibitory role in the Th1 and Th2 differentiation (Figure 1A). These conclusions are consistent with the results of Tumes et al. who showed that EZH2-deficient Th cells produce more IFN-γ during stimulation in the presence of IL-12, which induces Th1 polarization and more IL-4, IL-5, and IL-13 during stimulation in the presence of IL-4, which induces Th2 polarization (27). Moreover, in the same study was found that EZH2-deficient T cells express higher amounts of T-bet and Gata-3 (27), critical transcriptional factors for the Th1 and Th2 differentiation, respectively. Of note, Tumes et al. showed that, compared to WT cells, Th1 polarized EZH2-deficient T cells express higher levels of IL-4, IL-5, and IL-13 when exposed to Th2 conditions and Th2 polarized EZH2-deficient T cells express higher levels of IFN-γ when exposed to Th1 conditions (27), suggesting that EZH2-deficient cells demonstrate increased plasticity. The inhibitory role of EZH2 on Th2 differentiation was further confirmed by the finding that the transfer of oval-albumin (OVA)-specific EZH2-deficient Th2 cells in C57BL/6 mice was associated with the development of exaggerated allergic asthma characterized by increased eosinophilic inflammation and increased proportion of effector memory CD4^+^ T cells, which produced increased amounts of IL-4 and IL-5 compared to the transfer of WT Th2 cells (27). These findings provided compelling evidence that EZH2 inhibits the differentiation and plasticity of naïve T cells. Based on these observations, it can be hypothesized that EZH2 inhibitors might enhance immune responses. In this context, EZH2 inhibitors might promote IFN-γ-producing T cells and can be used in order to enhance anti-tumor immunity, while EZH2 activating agents might serve as promising therapies for the suppression of Th1- and Th2-dependent autoimmune diseases.

It should be noted that the results of the various groups regarding the role of EZH2 on Th1 and Th2 differentiation are not completely consistent (24–28). Specifically, whereas three investigator groups reported increased IFN-γ production from EZH2-deficient T cells cultured under Th1 polarizing conditions (25–27), Tong et al. reported the opposite observation (28). This study found that downregulation of EZH2 decreases the production of IFN-γ under Th1 skewing conditions, which is not affected by neutralization of IL-4 and is not associated with differences in Gata-3 levels, suggesting that EZH2 promotes Th1 differentiation *in vitro* independently of IL-4 and Gata-3 (28). The authors showed that loss of EZH2 promotes the development of a particular histone methylation signature for genes associated with Th1 differentiation such as *Ifng, Tbx21*, and *Stat4* (28). These investigators also reported that EZH2 increases the stability of T-bet (28), suggesting a novel role of EZH2 as an inducer of Th1 differentiation. Interestingly, enhancement of Th1 differentiation through EZH2 was associated with the development of T cell-mediated aplastic anemia in transgenic mouse models, suggesting that targeting EZH2 might be a possible therapeutic approach for the treatment of aplastic anemia (28). These results are in agreement with a study by Jacob et al. who found that chromatin binding of the EZH2 to cytokine promoters in CD4^+^ T cells is associated with induction of Th1 and Th2 cytokines, suggesting a positive role of EZH2 toward cytokine gene transcription in CD4^+^ T cells (29). However, it is possible that the reason for this discrepancy might be purely methodological. Specifically, it was observed that enhanced Th2 differentiation and increased production of IL-4 by EZH2-deficient cells opposes Th1 polarization (25–27). Notably, the only study that observed decreased IFN-γ production – a hallmark of Th1 polarization – did not report the method of naïve T cell purification prior to *in vitro* polarizing culture (28). Therefore, it is possible that different isolation methods and lower purification efficiency might have resulted in the diminished ability of naïve EZH2-deficient T cells to be polarized toward Th1 phenotype observed in this one study.

Although EZH2 is a known methyltransferase acting as epigenetic regulator, Su et al. highlighted a cytosolic function of this protein in mediating T cell receptor-induced actin polymerization and T cell differentiation (14). Subsequently, Gunawan et al. showed that EZH2 deficiency impairs integrin-dependent migration of leukocytes affecting the progression of multiple sclerosis in a mouse autoimmune model further supporting a critical extranuclear function of EZH2 in the development of inflammation and autoimmune disease (30).

### EZH2 and Effector T Cells

Apart from the differentiation of naïve CD4^+^ T cells, the function of the immune system also depends on the survival of differentiated immune cells. Zhang et al. found that EZH2 promotes the survival of differentiated effector T cells through inhibition of numerous apoptosis pathways, including Fas, TNFR1, DR4, and Mlk1 signaling (25) (Figure 1B). Consistent with this finding, Yang et al. showed that EZH2-deficient mice have decreased survival after intraperitoneal *Toxoplasma gondii* infection and this was associated with decreased numbers of IFN-γ producing CD4^+^ T cells in the peritoneal exudate (26). These observations suggest that EZH2 is critical for the generation of T effector cell responses *in vivo*, and the defects observed in EZH2-deficient mice were attributed to the decreased proliferation of EZH2 effector T cells. The conclusion that EZH2 is critical for the survival, proliferation, and activity of effector T cells was further supported by a study by He et al. who evaluated the role of EZH2 in the development of GVHD using a model of allogeneic bone marrow transplantation (31). Particularly, the authors showed that EZH2 is critical for the survival and proliferation of alloantigen-activated T cells and the development of GVHD in a major histocompatibility (MHC)-mismatched B6 anti-BALB/C mouse model. In that system, IFN-γ producing alloreactive T cells are significantly diminished in the absence of EZH2 (31). Of note, the deletion of EZH2 did not affect the anti-leukemic effect of donor T cells in this mouse model (31). This observation suggested that inhibition of EZH2 might be a reasonable approach to protect patients undergoing bone marrow transplantation from the development of GVHD without compromising the beneficial graft-versus-leukemia effect of allogeneic stem cell transplantation.

### EZH2 and T Regulatory Cells

While the studies presented above supported a critical role of EZH2 on Th1 and Th2 differentiation of naïve CD4^+^ T cells with very tentative therapeutic implications, the deletion of EZH2 has been associated with significant reduction of FOXP3 (25), suggesting that EZH2 may be critical for the activation of Tregs. FOXP3 co-localizes with EZH2 (32) and it has been shown that the absence of EZH2 is associated with impairment of iTreg differentiation *in vitro* (25, 27). These studies also indicated a possible positive role of EZH2 in the regulation of Treg differentiation, activation, and suppressive function (Figure 1C). Yang et al. evaluated the implication of EZH2 on Treg function and found that CD4-specific deletion of EZH2 in transgenic mice leads to significantly fewer naïve T cells and decreased FOXP3 expression in CD4 cells in the spleen and lymph nodes (26). Interestingly, the authors found that the addition of antibodies against IFN-γ and IL-4 reverses the impaired FOXP3 expression in EZH2-deficient T cells (26). This observation is consistent with the data of Zhang et al. showing that the FOXP3 reduction due to EZH2 deficiency is reversed in the presence of IFN-γ blocking antibody (25). These findings imply that the downregulation of FOXP3 in EZH2-deficient cells is likely mediated by aberrant production of cytokines, such as IFN-γ and IL-4. To expand these findings and understand their biological implications *in vivo*, Yang et al. studied the functional implications of EZH2 deficiency on the properties of Tregs and determined that EZH2-deficient Tregs failed to protect from the development of autoimmunity in a model of naïve T cell-mediated colitis *in vivo* (26).

Further highlighting the critical role of EZH2 in the activation of Tregs, DuPage et al. showed that, at the transcription level, EZH2 is the most abundant chromatin modifier induced by CD28 activation in naïve CD4^+^ T cells (33). This is consistent with previous reports supporting that the epigenetic state of Tregs is regulated by pathways related to the T cell receptor-mediated stimulation (34, 35). More importantly, Treg-specific EZH2 deletion renders Tregs phenotypically normal and functional *in vitro* but unable to properly maintain immune homeostasis *in vivo*, as supported by the fact that Treg.EZH2Δ/Δ mice presented numerous signs of autoimmunity, including weight and hairy loss and scaly tails (33). The authors showed that EZH2 is responsible for the maintenance of the repressive gene program after Treg activation, which is critical for the recruitment and function of Tregs at the site of inflammation (33). These results support an important role of EZH2 in the activity of Tregs, which may have significant implications not only in the context of autoimmune diseases and cancer.

### EZH2 and Regulation of Anti-Tumor Immunity

Despite the extensive research evaluating the role of EZH2 as a promoter of cancer progression through the induction of cell cycle and inhibition of cancer cell differentiation, the effects of EZH2 expression on the tumor immunity have not been fully appreciated. According to a recent publication by Peng et al., epigenetic alterations of cancer cells by inhibition of EZH2 and DNA methyltransferase 1 (DNMT1) in a mouse model of ovarian cancer resulted in increased expression of the Th1-type chemokines CXCL9 and CXCL10 in cancer cells leading to increased trafficking of effector T cells to the tumor site and decreased tumor volume (36). Conversely, increased expression of EZH2 and DNMT1 in ovarian tumors was associated with decreased infiltration of CD8^+^ T cells and worse prognosis (36) (Figure 1D). Furthermore, treatment of ovarian tumors with EZH2 and DNMT1 inhibitors increased the efficacy of tumor-associated antigen-specific CD8^+^ T cells in response to PD-L1 inhibition. A similar effect of EZH2 was observed in colorectal cancer, where targeting EZH2 in cancer cells augments the expression of CXCL9 and CXCL10 chemokines affecting the infiltration of the tumor by effector T cells (37). These results support the conclusion that the tumorigenic effects of histone modifications and DNA methylation in cancer cells may be mediated by alteration of immunity in the tumor microenvironment. This conclusion may lead to the introduction of novel therapeutic strategies to improve the efficacy of immunotherapy.

## Conclusion

Enhancer of zeste homolog 2 is a methyltransferase, member of the PRC2 complex acting as epigenetic regulator with critical implications in maintaining the gene signature particularly associated with cell cycle progression, proliferation, and differentiation. Recent studies suggest that EZH2 has significant implications in carcinogenesis and is a novel target in cancer therapeutics. While EZH2 inhibitors are currently under intense investigation, the implications of EZH2 in the regulation of T cell differentiation and activity are only now starting to be unraveled. Such effects of EZH2 are of particular interest because targeting T cells-specific EZH2 functions may have significant implications for the control of autoimmunity, alloreactivity, and anti-tumor immunity. Strikingly, EZH2 appears to function as a crosstalk regulator between cancer and T cells and modulation of EZH2 expression in cancer cells alters their ability to produce Th1-type chemokines and to attract CD8^+^ T effector cells, which mediate anti-tumor function especially in the context of checkpoint blockade immunotherapy. Importantly, EZH2 has activity not only on T effector cells but also on Tregs, rendering the therapeutic targeting of EZH2 a uniquely complex challenge. Further studies will be required to clarify the effects of EZH2 on the differentiation, survival, and function of Th, T effector, and Tregs in order to create novel therapeutic strategies in the area of autoimmune diseases, allogeneic transplantation, and cancer.

## Author Contributions

TK did the outline of the work and generated the main body of the manuscript and figures; AC wrote individual subsections of the manuscript; KB wrote subsections of the manuscript; LL provided comments in all sections and wrote additional points; and VB revised the manuscript and figures and provided input in all sections.

## Conflict of Interest Statement

The authors declare that the research was conducted in the absence of any commercial or financial relationships that could be construed as a potential conflict of interest.
