# Corrigendum: Regulation of T Cell Differentiation and Function by EZH2

**DOI:** 10.3389/fimmu.2016.00346

**Published:** 2016-09-14

**Authors:** Theodoros Karantanos, Anthos Christofides, Kankana Bardhan, Lequn Li, Vassiliki A. Boussiotis

**Affiliations:** ^1^Division of Hematology-Oncology, Beth Israel Deaconess Medical Center, Harvard Medical School, Boston, MA, USA; ^2^Department of Medicine, Beth Israel Deaconess Medical Center, Harvard Medical School, Boston, MA, USA; ^3^General Internal Medicine Section, Boston Medical Center, Boston University School of Medicine, Boston, MA, USA; ^4^Beth Israel Deaconess Cancer Center, Harvard Medical School, Boston, MA, USA

**Keywords:** T cells, T cell differentiation, T cell activation, tumor immunity, EZH2

The names of two authors were misspelled in the original article as Anthos Chistofides and Kankana Barhdan. The correct spelling of these names is Anthos Christofides and Kankana Bardhan.

The authors apologize for this. This error does not change the scientific conclusions of the article in any way.

The original article has been updated.

## Conflict of Interest Statement

The authors declare that the research was conducted in the absence of any commercial or financial relationships that could be construed as a potential conflict of interest.

